# Lumbar spine bone mineral density and trabecular bone score-adjusted FRAX, but not FRAX without bone mineral density, identify subclinical carotid atherosclerosis

**DOI:** 10.1007/s40618-021-01517-4

**Published:** 2021-02-12

**Authors:** J. Pepe, G. Della Grotta, R. Santori, V. De Martino, M. Occhiuto, M. Cilli, S. Minisola, C. Cipriani

**Affiliations:** Department of Clinical, Internal, Anesthesiology and Cardiovascular Sciences, (SCIAC), “Sapienza” University of Rome, Rome, Italy

**Keywords:** Atherosclerosis, Osteoporosis, Carotid intima media thickness, Trabecular bone score, FRAX

## Abstract

**Purpose:**

Osteoporosis and atherosclerosis share common risk factors. Aim of this study was to test if FRAX (which is an algorithm that can identify subjects at risk of fracture), without or with BMD values, also adjusted for trabecular bone score (TBS) was able to identify subclinical atherosclerosis, evaluated by measurement of carotid intima media thickness (cIMT ≥ 0.9 mm) as compared to DXA values.

**Methods:**

Ninety postmenopausal women underwent DXA measurement and cIMT evaluation. For each patient, the FRAX algorithm for major osteoporotic fracture (M) and for hip fracture (H) without BMD was computed, together with FRAX with BMD and TBS-adjusted FRAX. Serum levels of osteoprotegerin, sRANKL, and interleukin-6 were also measured.

**Results:**

There were no differences in anthropometric parameters and cardiovascular risk factors between subjects with cIMT ≥ 0.9 mm (35% of subjects, group A) compared to those with cIMT < 0.9 mm (group B). The prevalence of osteoporosis and FRAX BMD, TBS-adjusted FRAX both for M and H were higher in group A compared to group B. The best ROC curves to identify subjects with a cIMT ≥ 0.9 mm were: lumbar spine T-score, with a threshold of − 2.5 SD (area under the curve, AUC 0.64; *p* = 0.02) with a sensibility of 50% and a specificity of 76%; TBS-adjusted FRAX H with a sensibility of 50% and a specificity of 72% (AUC 0.64; *p* = 0.01 with a threshold of 3%). Interleukin-6 positively correlated with FRAX BMD H and M.

**Conclusions:**

FRAX without BMD does not identify subclinical carotid atherosclerosis, while lumbar spine T-score and TBS-adjusted FRAX H similarly detected it with higher specificity for T-score.

## Introduction

Recent meta-analyses showed that low bone mineral density (BMD) is independently associated with cardiovascular diseases [1, 2] and in particular with atherosclerosis [3]. However, there are many risk factors in common between these two conditions that need to be taken into account and can probably influence the strength of this association.

Preclinical atherosclerosis can be assessed by carotid intima media thickness (cIMT) measurement, a widely accepted marker of atherosclerosis and predictor of future vascular events [4]. Several studies reported significantly higher cIMT values in patients with osteoporosis compared to non-osteoporotic subjects, which were negatively correlated with BMD values [5–8].

To explain this association, a possible common pathophysiological mechanism underlying these diseases still needs to be found. However, both vascular calcification and bone metabolism share some bone derived proteins such as osteocalcin [9] and osteoprotegerin (OPG) [10, 11]. Moreover, low vitamin D levels have been linked to both cardiovascular disease and bone fragility [12, 13], also because it causes secondary hyperparathyroidism [14]. Finally, inflammation with high interleukin 6 level has been reported in both diseases [15, 16].

Although these molecular shared pathways are still areas of research and uncertainty, there are well-established clinical risk factors for osteoporosis and cardiovascular disease [17–20].These are age, sex and specific lifestyle habits, such as excessive alcohol intake, smoking, high body mass index (BMI) and glucocorticoid use [21]. Cardiovascular diseases and osteoporosis are both more frequent in some endocrinological or rheumatological diseases such as rheumatoid arthritis [21]. Also, bone fragility fractures have been associated with cardiovascular disease, in particular, with development of carotid atherosclerosis [22]. All of the above mentioned shared clinical risk factors regarding bone fragility and cardiovascular disease are the risk factors included in a computer-free algorithm, FRAX®, created to predict 10-year fracture risk in subjects ranging from 40 to 90 years of age. This algorithm also included the possibility to add the femoral neck bone mineral density (BMD) value or neck T-score [23]. A recent improvement of this tool was the possibility of adjusting FRAX values with the trabecular bone score (TBS), which is an indirect measure of bone quality, derived from the dual-energy X-ray absorptiometry (DXA) scan image of the lumbar spine with a specific software [24]. TBS has been associated with the risk of fractures independently of BMD [24].

To the best of our knowledge only one study has been carried out, showing a positive correlation between FRAX without BMD and a surrogate of peripheral atherosclerosis (brachial artery IMT) in a general population of men and women [25]. There are no studies which aimed to evaluate the performance of FRAX with or without BMD, or also with TBS-adjusted FRAX to identify patients with subclinical carotid atherosclerosis compared to DXA values alone. Aim of this study was to evaluate if FRAX, with or without BMD and also TBS-adjusted FRAX were able to identify postmenopausal women with subclinical atherosclerosis, evaluated with cIMT compared to DXA values alone. Several biochemical molecules that may explain the physiopathological link between atherosclerosis and osteoporosis such as OPG, soluble receptor activator of nuclear factor kappa-Β ligand (sRANKL), and interleukin-6,were also measured.

## Methods

### Patients

Between January 2017 and April 2020, 100 consecutive postmenopausal women were enrolled at the Bone Disease Unit of “Sapienza” University during their first visit for osteoporosis screening. The inclusion criteria for this study were age range from 50 to 85 years and being postmenopausal. Based on medical interviews, we excluded patients with neoplasia, presence of previous cardiovascular diseases and diabetes mellitus type 1 and 2. We did not measure serum glucose. None of the patients enrolled had ever undergone any treatment which could interfere with mineral metabolism (steroids, diuretics, thyroid hormones, anticonvulsant drugs, lithium, etc.). Furthermore, no patients reported a history of known secondary osteoporosis. Another exclusion criterium was an estimated creatinine clearance < 60 ml/min. We did not perform a lipid panel measurement; however, dyslipidemia was anamnestically evaluated on the basis of the latest blood examination (no later than 6 months) [26]. Hypertension was ascertained through blood pressure measurement and defined as systolic blood pressure > 140 mmHg or diastolic blood pressure > 90 mmHg.

All subjects gave written informed consent and the study was approved by the local hospital’s Research Ethics Committee.

### Laboratory investigations

Mineral metabolism was evaluated by measuring: serum total calcium (Ca), phosphorus (P), 25-hydroxyvitamin D [25(OH)D], parathyroid hormone (PTH) and creatinine (Cr), as previously described [27, 28]. Creatinine clearance was estimated by the formula of Cockcroft and Gault. We utilized the ELISA kits for the following measurements: bone isoenzyme of alkaline phosphatase [AC-20, IDS, UK, inter-assay coefficient of variation (CV) was 4.5% and intra-assay CV 5.9%], C-terminal telopeptide of collagen type I (AC-02F1, IDS, UK inter-assay CV 5%, intra-assay CV 6%), receptor activator of nuclear factor kappa-Β ligand (sRANKL, Biovendor, Czech Republic, inter-assay CV 7.2%, intra-assay CV 9%), OPG (Biovendor, Czech Republic, inter-assay CV 4.9%, intra-assay 7.1%) and Interleukin-6 (R&D System, Minneapolis, USA inter-assay CV 4%, intra-assay CV 6.3%).

### Radiological examination

Each subject underwent standardized spine lateral radiographs to detect vertebral deformity, defined according to Genant’s method. BMD of the lumbar spine (L1–L4), in the anterior–posterior projection and of the hip (femoral neck and total hip), was measured in each patient with DXA scan (Hologic QDR 4500, Hologic Inc., Waltham, MA, USA). Fractured vertebrae were excluded. The precision error of lumbar spine and total hip measurement was 1.3 and 1.7%, respectively [29]. DXA results of lumbar and/or neck site were classified according to the World Health Organization (WHO) diagnostic criteria for osteoporosis [normal (T-score ≥ 1 SD), osteopenic (T-score between − 1 and < -2.5 SD) and osteoporotic (T-score ≤ − 2.5.SD)]. TBS was computed, after fractured vertebrae were excluded, in the same regions as those used for lumbar spine BMD using TBS iNsight (version 3.0.2.0, Med-Imaps, Switzerland), as the mean value of the individual measurement for vertebrae L1–L4. The coefficient of variation for TBS was 1.8% and did not vary among the measured vertebrae.

### cIMT

The evaluation of cIMT was accessed using high-resolution B-mode ultrasound (Mindray, dc-80, x-insight) according to the protocols of the American Association of Echocardiography [30]. Early carotid atherosclerosis was defined as a mean cIMT ≥ 0.9 mm.

### FRAX

For each patient, the FRAX algorithm was computed for major osteoporotic fracture (M), i.e., clinical spine, forearm, hip and proximal humerus and for hip fracture alone (H), without BMD [31]. FRAX is a computer-based algorithm that provides the 10-year probability of fractures in men and women on the basis of classic risk factors (CRFs), such as age, sex, weight, height, previous fracture, parent hip fracture, current smoking, current glucocorticoid use, rheumatoid arthritis, secondary osteoporosis and alcohol use (> 3 units/day), alone, or by integration of CRFs with BMD hip T-score (FRAX BMD); finally, a FRAX computation was made, adjusted for trabecular bone score (TBS-adj FRAX).

### Statistical analysis

Results are presented as mean values ± 1 standard deviation (SD). Significance between means was assessed using the Student’s *t*-test. Frequencies were compared using chi-squared test. Pearson correlation coefficient was used to assess the relationship between continuous variables, in particular between cIMT and biochemical parameters, DXA values, as well as FRAX. Receiver operating characteristic (ROC) curves were designed to identify which of the parameters studied was best suited to predict the cIMT. For each ROC curve, specificity and sensitivity were calculated (95% confidence interval, CI). The statistical analyses were carried out using the Statistical Package for the Social Sciences software (release 20; SPSS Inc., Chicago, IL, USA). The results were considered significant when the probability value was less than 0.05% (*p* value < 0.05).

## Results

Ten patients were excluded based on the inclusion criteria, among the 100 subjects screened. Considering the 90 patients enrolled, we found 32 patients with a cIMT ≥ 0.9 mm (35% of the population, group A). There were no differences in anthropometric parameters between group A and those subjects with a cIMT lower than 0.9 (group B), as shown in Table 1. There were no differences in cardiovascular risk factors between group A and group B. Indeed, hypertension was found in approximately 31% of patients in group A compared to 25% in group B (*p* = 0.63). Ace-inhibitors were the most frequent drug prescribed, with no difference between the groups. Dyslipidemia was found in 25% of the subjects in group A and 18% in group B (*p* = 0.59). Statins were prescribed in 18% of subjects in group A and 12% in group B (*p* = 0.53). Approximately 8% of the patients in group B had both hypertension and dyslipidemia compared to 12% in group A (*p* = 0.71). Considering other traditional risk factors for atherosclerosis, both the alcohol consumption (> 3 units/day) and the current smoking habits did not different between group A and group B (Table 1).

**Table 1:** Mean ± SD or percentage of the parameters needed for FRAX computation in the sample divided according to cIMT

‘	cIMT ≥ 0.9	cIMT < 0.9
	Group A	Group B
Age (years)	61.53 ± 7.39	61.79 ± 8.89
Body mass index (kg/m^2^)	24.50 ± 4.04	24.19 ± 4.08
Parental hip fracture	22%	17%
Vertebral fractures	22%	15.5%
Peripheric fracture	15.6%	1.72%*
Glucocorticoid	0%	0%
Rheumatoid arthritis	0%	0%
Secondary osteoporosis	0%	0%
Alcohol > 3 units/day	12.5%	9%
Current smoking	31%	34%
Trabecular bone score	1.050 ± 0.14	1.110 ± 0.87
T-score neck	− 1.98 ± 0.88	− 1.47 ± 0.75*

*p < 0.05

A higher prevalence of osteoporosis was found in group A compared to group B (46.8 vs 22.4%, *p* = 0.03) and also a higher number of non-vertebral fractures (15.6 vs 1.72%, *p* = 0.02), as shown in Tables 1 and 2. Mean values of FRAX H and M, computed without BMD, did not differ between the two groups (Table 2). However, the mean FRAX values adding femoral neck T-score values, were statistically significantly higher in group A for hip (FRAX BMD H) and for major fracture (FRAX BMD M) compared to group B (Table 2). Also, mean values of TBS-adj FRAX were significantly higher in group A compared to group B for both hip (TBS-adj FRAX H) and for major fracture (TBS-adj FRAX M). In particular, considering the threshold of 3% to define a subject at high risk for hip fracture for TBS-adj FRAX H, half of the subjects in group A were above this threshold; the double compared to group B (50 vs 25%, *p* = 0.03).

**Table 2:** Mean ± SD or percentage of bone parameters and FRAX in the sample dived according to cIMT

‘	cIMT ≥ 0.9	cIMT < 0.9
	Group A	Group B
Osteoporosis (%)	46.8	22.4*
Osteopenia (%)	43.7	56.8
Normal (%)	9.3	20.6
T-score L1–L4	− 2.25 ± 1.24	− 1.67 ± 1.27*
T-score total	− 1.63 ± 0.97	− 1.11 ± 0.87*
FRAX M (%)	6.39 ± 4.72	6.5 ± 2.08
FRAX H (%)	1.84 ± 2.52	2.08 ± 2.92
FRAX BMD M (%)	8.17 ± 6.27	5.95 ± 4.21*
FRAX BMD H (%)	2.86 ± 3.5	1.51 ± 2.14*
TBS-adj FRAX M (%)	12.95 ± 9.6	8.79 ± 4.89**
TBS-adj FRAX H (%)	5.36 ± 6.27	2.39 ± 2.20**

*H* hip, *M* major fractures, *TBS-adj* trabecular bone score adjusted, *BMD* bone mineral density; *p < 0.05, **p < 0.001

Mean values of biochemical parameters were not statistically different between the two groups (Table 3). The number of patients with serum levels of 25(OH) vitamin D less than 20 ng/ml was higher in group A compared to group B, but this difference was not statistically significant (40 vs 27%, *p* = 0.24). Among patients in group A, patients with vitamin D < 20 ng/ml had higher mean creatinine values than those with vitamin D > 20 ng/ml (0.85 ± 0.13 vs 0.73 ± 0.09 mg/dl, *p* = 0.004).

**Table 3:** MeanSD of the principle biochemical parameters of the sample divided according to cIMT

	cIMT ≥ 0.9	cIMT < 0.9
	Group A	Group B
Creatinine (mg/dl)	0.76 ± 0.13	0.82 ± 0.15
Calcium (mg/dl)	9.34 ± 0.36	9.36 ± 0.48
Phosphorus (mg/dl)	3.65 ± 0.60	3.73 ± 3.65
25(OH)D (ng/ml)	23.69 ± 11.61	24.02 ± 8.19
PTH (ng/l)	24.93 ± 8.73	24.02 ± 8.19
BALP (U/l)	12.17 ± 4.33	13.08 ± 5.49
CTX (ng/ml)	0.56 ± 0.27	0.55 ± 0.26
RANKL (pmol/l)	233.5 ± 144.36	203.76 ± 162.31
OPG (pmol/l)	8.20 ± 2.55	9.56 ± 4.09
RANKL/OPG	25.8 ± 21.58	32.41 ± 25.47
Interleukin-6 (pg/ml)	2.0 ± 2.08	2.11 ± 1.61

*25(OH)D* vitamin D, *PTH* parathyroid hormone, *BALP* bone isoenzyme of alkaline phosphatase, *CTX* C-terminal Telopeptide of collagen type I, *OPG* osteoprotegerin, *RANKL* receptor activator of nuclear factor kappa-Β ligand

Values of cIMT were neither associated with age nor with estimated creatine clearance. Values of cIMT were negatively associated with T-score at lumbar spine (*r* = − 0.25, *p* = 0.025) neck (*r* = − 0.28, *p* = 0.006) and total hip (*r* = − 0.27, *p* = 0.012). Moreover, values of cIMT were negatively associated with TBS (*r* = − 0.27, *p* = 0.010). A positive association were found between values of cIMT and FRAX BMD M (*r* = 0.24, *p* = 0.021), with FRAX BMD H (*r* = 0.25, *p* = 0.016), with TBS-adj FRAX M (*r* = 0.32, *p* = 0.002) and with TBS-adj FRAX H (*r* = 0.34, *p* = 0.001).

Both FRAX M and FRAX H were negatively associated with estimated creatine clearance (*r* = − 0.28, *p* = 0.03; *r* = − 0.29, *p* = 0.02, respectively).

Receiver operating characteristic (ROC) curves showed a high specificity for both neck and total hip T-score value (with a threshold of − 2.5 SD), but showed a low sensibility in identifying patients with cIMT ≥ 0.9 mm (Table 4). The ROC curve of FRAX H and M, of FRAX BMD M showed that none of these parameters was able to identify subjects with subclinical atherosclerosis (all *p* > 0.05, Table 4). Also, TBS values were not able to identify subjects with subclinical atherosclerosis (*p* > 0.05). The best ROC curve was for lumbar spine T-score. Considering a T-score threshold -2.5 SD, it was possible to identify subjects with a cIMT ≥ 0.9 mm, with a sensibility of 50% and with a specificity of 76% (area under the curve, AUC 0.64; 95% confidence interval (CI) 0.52–0.76, *p* = 0.02). Similarly, TBS-adj FRAX H had a sensibility of 50%, and a specificity of 72% (AUC 0.64; 95% CI 0.52–0.77, *p* = 0.01, with a threshold of 3%). Furthermore, TBS-adj FRAX M had a low sensibility and a high specificity compared to TBS-adj FRAX H, as shown in Table 4.

**Table 4:** Area under the curve (AUC), sensibility and specificity for ROC curves that identify subjects with cIMT ≥ 0.9 mm (*p* value < 0.05)

	Sensibility (%)	Specificity (%)	AUC (95%CI)	*p* value
T- score lumbar spine	50	76	0.64 (0.52–0.76)	0.02
T- score femoral neck	40.6	89.6	0.67 (0.54–0.79	0.007
T- score total hip	23.3	92.8	0.65 (0.53–0.78)	0.01
FRAX BMD H	31.2	84	0.63 (0.51–0.76)	0.03
TBS adj-FRAX BMD H	50	72	0.64 (0.52–77)	0.01
TBS adj-FRAX BMD M	15.6	94.8	0.63 (0.50–0.75)	0.04

For T-score values, the threshold was set at − 2.5

For FRAX BMD H and TBS adj-FRAX H, the threshold was set at 3%

For TBS adj-FRAX BMD M, the threshold was set at 20%

In the sample as a whole, values of cIMT were not statistically significantly associated with the biochemical parameters measured. However, both osteoprotegerin and interleukin-6 positively correlated with age (*r* = 0.23, *p* = 0.045; *r* = 0.27, *p* = 0.019, respectively), while the RANKL/OPG ratio correlated negatively (*r* = − 0.24, *p* = 0.04). Interleukin-6 positively correlated with FRAX M (*r* = 0.28, *p* = 0.01) and FRAX H (*r* = 0.23, *p* = 0.04), FRAX BMD M (*r* = 0.25, *p* = 0.002) and FRAX BMD H (*r* = 0.23, *p* = 0.04) and TBS-adj FRAX M (*r* = 0.23, *p* = 0.04).

## Discussion

To the best of our knowledge, we have shown, for the first time, that the FRAX algorithm (without BMD) does not identify subclinical atherosclerosis in postmenopausal women without known cardiovascular diseases. However, when adding BMD values we observed a significant capability in identifying subclinical atherosclerosis. Moreover, TBS-adj FRAX H has a similar ability in identifying subclinical atherosclerosis comparted to T-score lumbar spine alone. The risk of developing atherosclerosis increases, with advancing age; however, in our population we have not observed an association between age and cIMT, probably because the majority of the population has a normal value of cIMT. Indeed, our results showed that DXA values are independently associated with cIMT, because adding all of the clinical risk factors such as age, BMI, etc. included in FRAX did not increase the ability to identify subclinical atherosclerosis.

Although both lumbar spine T-score and TBS-adj FRAX H do not have a high sensibility in identifying subclinical atherosclerosis, they do have a good specificity. The best specificity above 90% was for hip T-score and TBS-adj FRAX BMD H at the expense of sensibility. This result is in line with previous literature that showed significant association between neck T-score in postmenopausal women and cIMT values [8]. The only study that showed that FRAX, without BMD, was associated with brachial IMT included patients at high risk of cardiovascular disease. In that study, approximately a third of the population involved men and women with diabetes mellitus, 70% with hypertension, and more than 30% with a history of previous stroke or coronary artery diseases [25]. This population was very different from the population enrolled in our study, where previous cardiovascular disease and diabetes were exclusion criteria.

For the first time, we also included TBS in the FRAX computation, for detecting subclinical atherosclerosis. It should be noted that in our sample TBS correlates with cIMT but TBS was not able to identify patients with cIMT ≥ 0.9 mm. However, in particular patients, such as those affected by diabetes mellitus type 2, TBS values were better associated with cIMT than BMD [32]. In diabetes mellitus, TBS has been found to be lower compared to BMD values, indicating a poor bone quality [32]. However, patients enrolled in our study were not affected by secondary osteoporosis, where TBS might be a more sensitive parameter of bone health.

We found a high percentage of patients with 25(OH)D lower than 20 ng/ml in the group with subclinical atherosclerosis, which is not surprisingly due to the frequent hypovitaminosis D in postmenopausal women in this age group [33]; this finding was not statistically significant, even though previous studies have indicated an association between low vitamin D levels and atherosclerosis [34]. Low vitamin D is associated with higher serum PTH levels, as has been found in a previous study in subjects with a higher cIMT [35]. However, in our study, we did not find a significant difference in the PTH values between group A and B.

Our study is subject to a few limitations. We did not measure serum glucose levels, considering that an interplay between glucose metabolism and bone, as well as atherosclerosis, is well documented [36, 38]. However, diabetes was one of the exclusion criteria, but because we did not measure serum glucose it is not possible to exclude that we also enrolled patients with fasting glucose and/or impaired glucose tolerance. Lipids were not biochemically assessed, although it should be noted that we did not find differences between patients with or without carotid subclinical atherosclerosis, when a previous diagnosis of dyslipidemia were specifically asked to patients. Adipokines were also not measured, which have been shown to be associated with both bone metabolism and atherosclerosis [39, 40]. Moreover, we only studied women. A recent study, conducted mainly in males in Taiwan, showed a correlation between the coronary artery calcification score and the FRAX with BMD. However, they enrolled a different population compared to that of our study. In fact, 11.1% were diabetic and 17.3% had risk factors of secondary osteoporosis [41].

In conclusion, our study found that FRAX without BMD was not able to identify subclinical carotid atherosclerosis; the presence of lumbar spine osteoporosis should suggest subclinical carotid atherosclerosis in a population of postmenopausal women without a previous diagnosis of cardiovascular diseases, independently of an age effect. Although TBS adj-FRAX H showed very similar performance compared to lumbar spine T-score for the identification of subjects with subclinical carotid atherosclerosis, it does not provide additional information compared to using lumbar spine T-score alone.

Our study highlights the interplay between bone and vascular system beyond common identifiable clinical risk factors. The common pathophysiological mechanism, that independently of clinical risk factors underlie this association, should be better evaluated.
